# Human Cytochrome P450 Cancer-Related Metabolic Activities and Gene Polymorphisms: A Review

**DOI:** 10.3390/cells13231958

**Published:** 2024-11-26

**Authors:** Innokenty M. Mokhosoev, Dmitry V. Astakhov, Alexander A. Terentiev, Nurbubu T. Moldogazieva

**Affiliations:** 1 Independent Researcher, 108815 Moscow, Russia; imokhosoev@mail.ru; 2 Department of Biochemistry, I.M. Sechenov First Moscow State Medical University (Sechenov University), 119991 Moscow, Russia; astakhov_d_v@staff.sechenov.ru; 3 Department of Biochemistry and Molecular Biology, N.I. Pirogov Russian National Research Medical University, 117997 Moscow, Russia; terentjev_aa@rsmu.ru

**Keywords:** CYPs, bioactivation vs. detoxification, drugs as substrates, inducers, and inhibitors, nuclear receptors and efflux transporters, gene polymorphisms, hormone-dependent cancers

## Abstract

Background: Cytochromes P450 (CYPs) are heme-containing oxidoreductase enzymes with mono-oxygenase activity. Human CYPs catalyze the oxidation of a great variety of chemicals, including xenobiotics, steroid hormones, vitamins, bile acids, procarcinogens, and drugs. Findings: In our review article, we discuss recent data evidencing that the same CYP isoform can be involved in both bioactivation and detoxification reactions and convert the same substrate to different products. Conversely, different CYP isoforms can convert the same substrate, xenobiotic or procarcinogen, into either a more or less toxic product. These phenomena depend on the type of catalyzed reaction, substrate, tissue type, and biological species. Since the CYPs involved in bioactivation (CYP3A4, CYP1A1, CYP2D6, and CYP2C8) are primarily expressed in the liver, their metabolites can induce hepatotoxicity and hepatocarcinogenesis. Additionally, we discuss the role of drugs as CYP substrates, inducers, and inhibitors as well as the implication of nuclear receptors, efflux transporters, and drug–drug interactions in anticancer drug resistance. We highlight the molecular mechanisms underlying the development of hormone-sensitive cancers, including breast, ovarian, endometrial, and prostate cancers. Key players in these mechanisms are the 2,3- and 3,4-catechols of estrogens, which are formed by CYP1A1, CYP1A2, and CYP1B1. The catechols can also produce quinones, leading to the formation of toxic protein and DNA adducts that contribute to cancer progression. However, 2-hydroxy- and 4-hydroxy-estrogens and their O-methylated derivatives along with conjugated metabolites play cancer-protective roles. CYP17A1 and CYP11A1, which are involved in the biosynthesis of testosterone precursors, contribute to prostate cancer, whereas conversion of testosterone to 5α-dihydrotestosterone as well as sustained activation and mutation of the androgen receptor are implicated in metastatic castration-resistant prostate cancer (CRPC). CYP enzymatic activities are influenced by *CYP* gene polymorphisms, although a significant portion of them have no effects. However, *CYP* polymorphisms can determine poor, intermediate, rapid, and ultrarapid metabolizer genotypes, which can affect cancer and drug susceptibility. Despite limited statistically significant data, associations between *CYP* polymorphisms and cancer risk, tumor size, and metastatic status among various populations have been demonstrated. Conclusions: The metabolic diversity and dual character of biological effects of CYPs underlie their implications in, preliminarily, hormone-sensitive cancers. Variations in CYP activities and *CYP* gene polymorphisms are implicated in the interindividual variability in cancer and drug susceptibility. The development of CYP inhibitors provides options for personalized anticancer therapy.

## 1. Introduction

Cancer, which is an intricate, heterogeneous, and multifactorial disease, is a major cause of death, causing life expectancy to decline worldwide. In total, approximately 19.3 million new cancer cases and almost 10 million cancer-related deaths were estimated by the International Agency for Research on Cancer all over the world in 2020 [1]. Moreover, cancer incidence and mortality rates are rapidly increasing due to an aging and growing population and the emergence of new risk factors for cancer [2]. The majority of new cases and cancer-related mortalities in both sexes have been attributed to the following cancer types: breast cancer (11.7 and 6.9%, respectively), lung cancer (11.4 and 18%, respectively), prostate cancer (7.3 and 3.8%, respectively), colorectal cancer (10.0 and 9.4%, respectively), and liver cancer (4.7 and 8.3%, respectively) [3].

Cancer cells are strongly dependent on their microenvironment and are regulated by a variety of endogenous and exogenous stimuli [4,5,6]. Both reprogrammed and oxidative metabolisms are maintained by cancer cells due to their metabolic plasticity [7,8]. A variety of metabolites have been implicated in cancer initiation and progression through their capability to interact with macromolecules such as DNA, proteins, and lipids [9,10,11]. This leads to the formation of toxic adducts, which cause disruptions in cellular signaling pathways, cellular dysfunction, and, ultimately, cell death.

Cytochromes P450 (CYPs) belong to the class of oxidoreductase enzymes with mono-oxygenase activity and the capability to metabolize a wide diversity of chemicals [12]. On the basis of sequence homology, the human CYP superfamily has been divided into 18 families, including 44 subfamilies and 57 isoforms, each of which is encoded by an individual gene. The currently accepted nomenclature of CYPs contains the abbreviation CYP followed by a number indicating a family (for example, CYP1 and CYP2), after which a capital letter points to a subfamily (for example, CYP3A and CYP11B), and another number specifies an isoform (for example, CYP3A4 and CYP105D5) [13].

Human CYPs are expressed in the liver; however, the extrahepatic expression of some isoforms in the lungs, prostate, kidneys, adrenal glands, and placenta has been reported [14]. Inside the cell, they are localized either in the inner membrane of the mitochondria or in the membranes of the endoplasmic reticulum (ER) [15]. The expression and abundance of different hepatic CYP isoforms have been shown to change during human developmental stages and lifetime [16]. Additionally, variations in the organ-, sex-, and ethnicity-specific expression of human CYPs have also been reported [17,18,19].

The most prominent human CYPs include the CYP1–CYP4 families, which are involved in a huge number of metabolic processes due to their high expression levels and broad spectrum of substrate specificities [20,21]. They have been implicated in the biosynthesis of various biologically active compounds and in both the bioactivation and inactivation of procarcinogens and xenobiotics. CYP3A4, CYP2C8, CYP2C9, CYP2D6, and CYP1A2 have been identified as key players in the metabolism of drugs, including those used in clinical practice [22]. Moreover, the crucial roles of CYP11, CYP17, and CYP19 family members—specifically, CYP11A1, CYP11B1, CYP11B2, CYP17A1, and CYP19A1—in the biosynthesis of steroid hormones, androgens, and estrogens are well-established [23,24]. All of these metabolic activities of CYPs proceed with the involvement of various redox partners, types of reactions, and intermediates [25,26].

Reactions catalyzed by CYP enzymes belong to phase I reactions and can be extremely diverse, including hydroxylation, epoxidations, dealkylation, desulfuration, dearylation, and deamination [27]. Normally, products of phase I reactions can further undergo conjugation, mostly with glucuronic acid, sulfuric acid, acetic acid, and glutathione (GSH), via phase II reactions [28]. Conjugation with the above-mentioned substances can occur with the involvement of UDP-glucanosyltransferases (UGTs), sulfotransferases (SULTs), *N*-acetyltransferases (NATs), and glutathione *S*-transferases (GSTs) [29]. Phase I and II reactions are part of the CYP-catalyzed biotransformation process, which yields polar compounds with increased water solubility, yet often preserving their activity. Such biotransformation serves to facilitate the excretion of waste and toxic compounds from an organism by the kidneys and the intestine.

However, the metabolites of phase I reactions such as procarcinogen and xenobiotic bioactivation can interact with DNA and proteins to yield toxic adducts, and this underlies their implication in cancer initiation, progression, and metastasis [30]. Due to the ability to activate prodrugs, they are involved in chemoprevention, whereas, due to the involvement in anticancer drug metabolism, CYPs can serve as targets in anticancer therapy [31]. Furthermore, various types of *CYP* gene polymorphisms such as single-nucleotide polymorphisms (SNPs) and copy number variations (CNVs) have been implicated in differences in an individual’s enzyme activity and anticancer drug response [32].

Several hundreds of variant alleles have been identified for genes encoding detoxifying and drug-metabolizing CYPs in humans [33,34]. The extensive genetic diversity of these CYP enzymes has been suggested to contribute to survival in the conditions of a changing environment and dietary restrictions [35]. However, substantial interindividual variations in *CYP* gene expression have been shown to exert only a limited or almost no effect on the metabolic activities of CYP enzymes. Indeed, Gao et al. have found 26 gene polymorphism sites in human liver microsomal *CYP* genes, and polymorphisms in only half of them including *CYP2A6*, *CYP2B6*, *CYP2C9*, *CYP2D6*, and *CYP3A4/CYP3A5* influence, to some extent, the enzyme catalytic activity [36]. Liu et al. have also identified that the interindividual variations in CYP mRNA expression levels are greater than in the enzyme activities, except for CYP2C19 [37]. This phenomenon is ethnicity-dependent; for example, as shown in the latter study, Hispanics had higher levels of CYP2C8 activity and CYP2B6, CYP2C9, and CYP2C19 mRNA expression than Caucasians, whereas African Americans had a low CYP2D6 mRNA expression level. Such variations in *CYP* gene expression and the enzyme activity affect the disease and drug susceptibilities among various populations [38].

In our review, we discuss the latest advancements in elucidating the roles of metabolic activities and gene polymorphisms of human CYP in cancer initiation, progression, and metastasis. We highlight that CYPs are implicated, mostly, in hormone-dependent cancers and liver injury and hepatocarcinogenesis. Therefore, we discuss the roles of various human CYP families in steroid hormone biosynthesis, xenobiotic detoxification, procarcinogen activation, and anticancer drug metabolism. In this context, we emphasize that CYPs exert both detoxifying and bioactivation capabilities, which reflect the diversity of human CYP metabolic activities. Such a metabolic diversity and dual character of biological effects dictate the limited number of cancer types, in which CYPs are implicated. We discuss the ethnicity-dependent *CYP* gene polymorphisms and highlight that only limited statistically significant data have been obtained by various research groups on the implication of *CYP* gene polymorphisms in cancer.

## 2. Metabolic Activities of Human CYPs

### 2.1. Biosynthesis of Steroid Hormones

The first studies on the mono-oxygenase activity of bovine adrenocortical microsomes, specifically the C21 hydroxylation of 17α-hydroxyprogesterone in the pathway of steroid biosynthesis from cholesterol, were conducted by Cooper et al. at the beginning of the 1960s [39,40]. Later, between the mid-1970s and early 1980s, it was shown that cytochrome P450 from rat testicular microsomes has 17α-hydroxylase and 17,20-lyase activities [41,42]. Afterwards, the involvement of a variety of redox partners in CYP-catalyzed reactions has been elucidated. In particular, Nakajin and Hall demonstrated that glucocorticoid and androgen biosynthesis in neonatal pig testis occurs due to both 17α-hydroxylase and 17,20-lyase activities, which require NADPH and P-450 reductase flavoprotein as redox partners [43].

**Figure 1:**
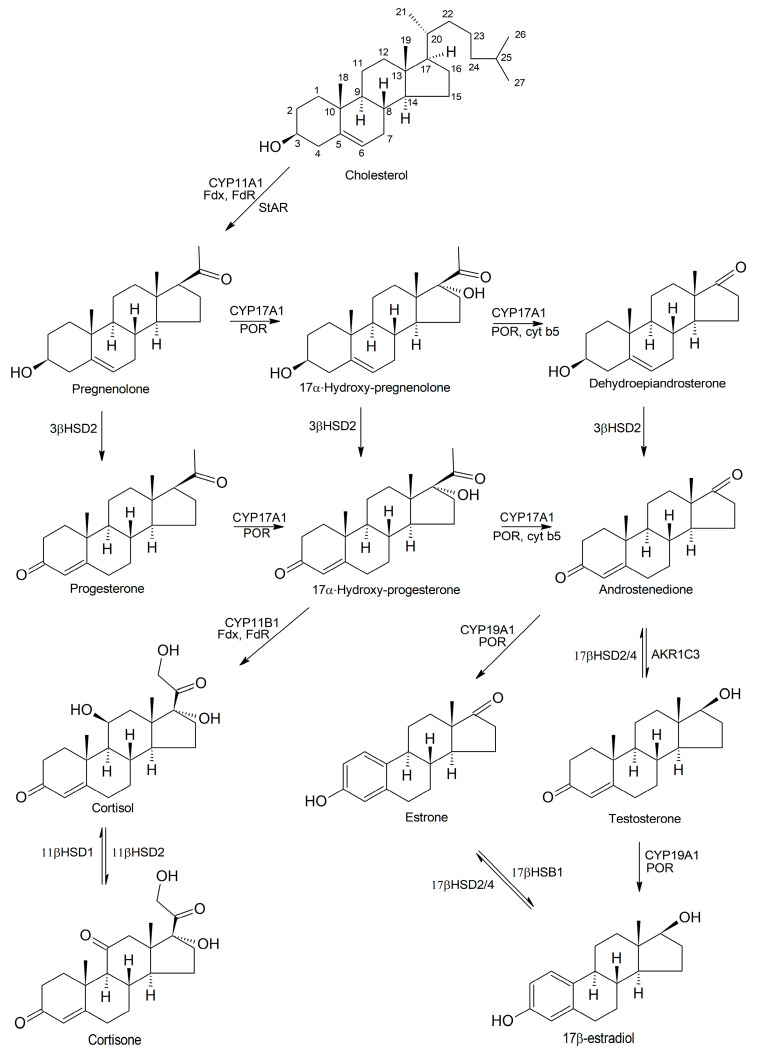
Steroid hormone biosynthesis. The side chain of cholesterol is initially oxidized and cleaved by CYP11A1 with the participation of its redox partners, Fdx and FdR, and StAR to yield pregnenolone (P5). In the ∆4 pathway, P5 undergoes oxidation of the hydroxyl group at C3 by 3βHSD2 to yield progesterone, whereas, in the ∆5 pathway, CYP17A1 hydroxylates P5 at C17 to produce 17α-OHP5, which is further converted to DHEA and androstenedione, both C19 androgen and C18 estrogen precursors. CYP19A1 converts androstenedione to estrone and testosterone to 17β-estradiol whereas CYP11B1 and CYP11B2 are essential for corticosteroid biosynthesis.

Currently, the steroid hormone biosynthesis pathways are well-studied. They are started from the oxidation and cleavage of the cholesterol side chain by CYP11A1 (P450scc), and this yields the C21-steroid, pregnenolone (P5) (Figure 1) [44]. This reaction comprises three sequential steps: (i) the oxidation of cholesterol at C22, (ii) the hydroxylation of the obtained product at C20, and (iii) the final cleavage of a bond between C20 and C22. For this reaction, cholesterol should be transported from the outer membrane to the inner membrane of the mitochondria, a process facilitated by the steroidogenic acute regulatory (StAR) protein [45]. Additionally, CYP11A1 requires redox partners including ferredoxin (Fdx), an iron-sulfur [2Fe-2S] cluster protein, and ferredoxin reductase (FdR), a mitochondrial flavoprotein [46].

P5 undergoes further conversions to androstenedione (∆4-dione) through either the ∆4 or ∆5 pathways, and the central roles of CYP17A1 in the P5 conversions in mammals are well-established. In the ∆4 pathway, P5 undergoes oxidation at its C3-hydroxyl group to a keto-group and a double bond switch from the B ring to the A ring, yielding progesterone (P4) in a reaction catalyzed by microsomal 3β-hydroxysteroid dehydrogenase 2 (3βHSD2) [47]. In the ∆5 pathway, P5 is hydroxylated at C17 to yield 17α-hydroxy-pregnenolone (17α-OHP5), which is further converted to dehydroepiandrostenedione (DHEA) due to the C17–C20 side chain cleavage [48,49]. These two reactions are catalyzed by CYP17A1 and require redox partners, either cytochrome P450 oxidoreductase (POR) or both POR and cytochrome *b5* (cyt *b5*) for electron transportation [50]. Androstenedione is a precursor of both estrogens and androgens, and all intermediates of both ∆4 and ∆5 pathways as well as CYP17A1 enzyme can serve as drug targets in the treatment of hormone-sensitive cancers such as prostate cancer (PCa) and breast cancer (BC) [51].

Further, the keto-group at C17 in androstenedione is reversibly reduced to a hydroxyl group to yield testosterone. This conversion is catalyzed by aldo-keto reductase 1C3 (AKR1C3), and can be reversed by 17β-hydroxysteroid dehydrogenase 2/4 (17βHSD2/4) [52]. CYP19A1, also known as aromatase, is another CYP superfamily enzyme that plays critical roles in estrogen biosynthesis and requires POR as a redox partner. It is expressed in the granulosa cells and corpora lutea of the ovary, Leydig and Sertoli cells of the testis, and other non-gonadal tissues such as placenta, brain, liver, and vascular smooth muscle [53]. CYP19A converts androstenedione to estrone, 16-hydroxytestosterone to estriol, and testosterone to 17β-estradiol.

Other steroid hormones include glucocorticoids synthesized in the *zona fasciculata* of the adrenal gland with the involvement of CYP11B1 (11β-hydroxylase) and mineralocorticoids produced in the outer *zona glomerulosa*, which expresses CYP11B2 (aldosterone synthase) [54]. CYP11B1 hydroxylates the 17α-OHP at C11 to produce cortisol, which is further converted to cortisone via the oxidation of the 11-hydroxyl group to a keto-group by 11βHSD1. CYP11B1 requires Fdx and FdR as the redox partners for glucocorticoid biosynthesis [55]. The soluble [2Fe-2S] cluster protein adrenodoxin (Adx), which belongs to ferredoxins, and adrenodoxin reductase (AdR), the membrane-bound NADPH-dependent flavoprotein, serve as CYP11B2 redox partners [56,57].

### 2.2. Detoxification vs. Bioactivation of Xenobiotics

Members of mammalian CYP1–CYP4 families are the most prominent CYPs involved in both detoxifying and bioactivating procarcinogens, drugs, and xenobiotics [58]. Rendic and Guengerich have found that these CYPs are more actively involved in xenobiotic metabolism than other enzymes including aldo-keto reductases (AKRs), flavin-containing mono-oxygenases (FMOs), and monoamine oxidase (MAO) [59]. The authors have assessed the reactions of metabolism of all chemicals, dividing them into drugs, physiological compounds (hormones, vitamins, and bile acids), and general chemicals (environmental and industrial pollutants). This has allowed the identification that 20% of reactions of xenobiotic metabolism can be attributed to the activities of CYP3A4, 10% to CYP1A2 and CYP2D6 each, 9% to CYP2C9, 8% to CYP2C8, 7% to CYP1A1, and 5% to CYP2B6 and CYP2E1 each. They also found that FMOs, MAO, and AKRs are collectively involved in only 5% of xenobiotic detoxification reactions.

**Figure 2:**
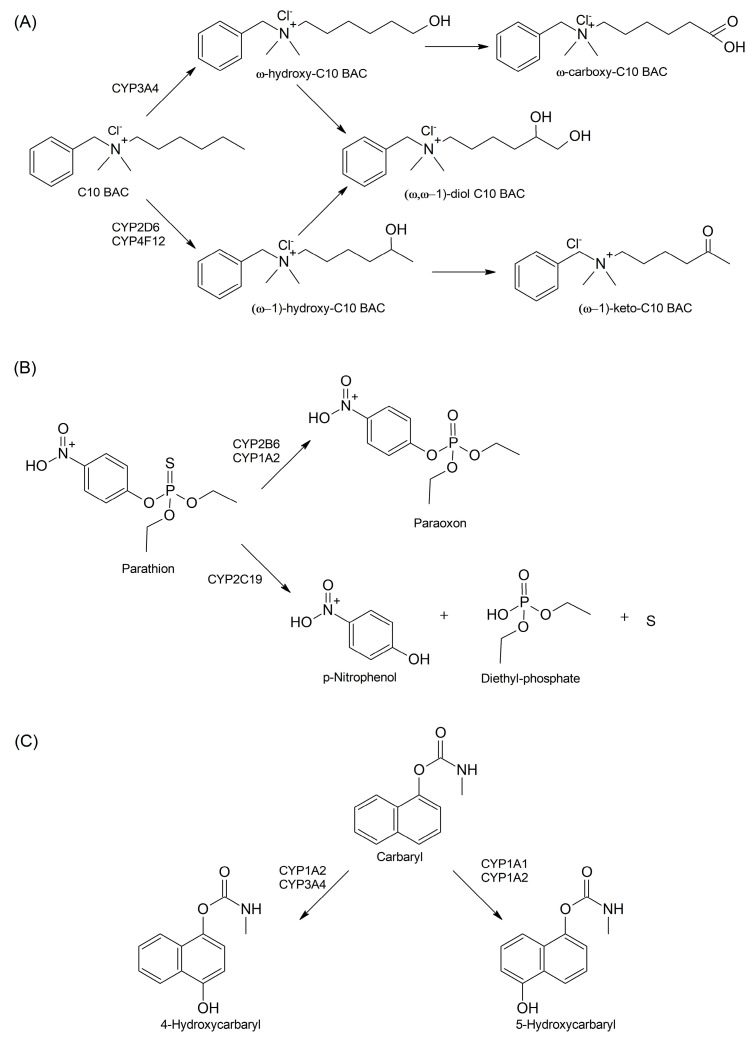
The implication of CYPs in detoxification versus bioactivation of xenobiotics in the liver. (**A**) CYP3A4, CYP2D6, and CYP4F12 catalyze the bioactivation of C10 benzalkonium chloride (C10 BAC) via the ω-oxidation reactions, and their major products include ω-hydroxy-, (ω − 1)-hydroxy-, (ω, ω − 1)-diol-, (ω − 1)-ketone-, and ω-carboxylic acid metabolites. (**B**) Parathion is activated to diethyl 4-nitrophenyl phosphate (paraoxon) by CYP1A2 and CYP2B6; however, CYP2C19 favors inactivation via oxidative cleavage of parathion to p-nitrophenol and diethyl-phosphate. (**C**) CYP1A1 and CYP1A2 catalyze the activation of carbaryl to 5-hydroxycarbaryl whereas CYP1A2 and CYP3A4 activate carbaryl to 4-hydroxycarbaryl.

The general chemicals metabolized by CYPs comprise hundreds of compounds. including alkyl dimethyl benzyl ammonium chlorides. also known as benzalkonium chlorides (BACs) [60]. BACs belong to common quaternary ammonium compounds (QACs), which exert cytotoxic effects and are hazardous to human health [61]. Recombinant CYP3A4, CYP2D6, and CYP4F12 have been shown to metabolize a significant portion of BACs to produce major liver microsomal metabolites [62]. The more lipophilic, longer-chain BACs demonstrated greater metabolic stability, which changed as follows: C10 < C12 < C14 < C16. The conversions of C10 BAC led to the formation of the most cytotoxic compounds, primarily through ω-oxidation reactions. The major metabolites of CYP3A4-catalyzed reactions include ω-hydroxy-C10 BAC, which is further converted to ω-carboxy- and (ω, ω − 1)-diol-derivatives. On the other hand, the main metabolites of reactions catalyzed by CYP2D6 and CYP4F12 are (ω − 1)-hydroxy-, and (ω − 1)-keto-derivatives (Figure 2A) [63].

Other groups of general chemicals comprise organophosphates and carbamates, which are used as pesticides and cause acute toxicity resulting from the inhibition of serine esterase enzymes including acetylcholinesterase (AChE) [64]. AChE catalyzes the breakdown of the neurotransmitter acetylcholine, and, therefore, AChE inhibition causes the excessive stimulation of both nicotinic and muscarinic acetylcholine receptors in the central and peripheral nervous systems. Chemically, organophosphates are modified esters of phosphoric acid and can include phosphorothioates containing a P=S group and phosphorodiamidates containing a P-N group. They are oxidatively activated via the production of corresponding oxons containing a P=O group [65]. Indeed, parathion, chlorpyrifos, malathion, and disulfoton are known to become more toxic than their parent compounds via CYP-catalyzed metabolic activation (Figure 2B). For example, parathion is metabolized to diethyl 4-nitrophenyl phosphate (paraoxon), which is a potent AChE inhibitor used as a parasympathomimetic drug [66]. However, menadione (methyl-1,4-naphthoquinone, and vitamin K3) inhibits the conversion of parathion to paraoxon by human liver microsomes and recombinant CYP1A2, CYP2B6, and CYP3A4 [67].

However, it has been reported that organophosphates can be converted to less toxic compounds depending on the CYP isoforms involved in their metabolism. Indeed, Foxenberg et al. have shown that CYP1A2, CYP2B6, CYP2C9, CYP2C19, CYP3A4, CYP3A5, and CYP3A7 with varying activity metabolize parathion and all of these isoforms except for CYP2C9 can metabolize chlorpyrifos [68]. These CYP isoforms can catalyze either the bioactivation or detoxification of both parathion and chlorpyrifos depending on the reaction type involved. Oxidative desulfuration yields oxon derivatives, thereby causing bioactivation, whereas dearylation via hydrolytic cleavage results in inactivation. Chlorpyrifos undergoes hydrolytic cleavage to yield 3,5,6-trichloro-2-pyridinol (TCP), whereas parathion is detoxified via degradation to p-nitrophenol (PNP) (Figure 2B). CYP2B6 had a great potential to produce oxon derivatives with low K_m_ and high V_max_ values, whereas CYP2C19 showed the highest V_max_/K_m_ ratio in PNP and TCP formation. The conversions of organophosphorus pesticides to either more or less toxic metabolites depend on *CYP* gene polymorphisms as shown in the CYP2B6-catalyzed metabolism of chlorpyrifos [69].

Commonly used carbamate pesticides include carbaryl, carbofuran, methiocarb, and aminocarb, which, unlike organophosphates, reversibly inhibit AChE [64]. It has been shown that methiocarb is activated via oxidation to sulfoxide and then to sulfone by FMO1 and CYP2C19 [70], Methiocarb is inactivated via hydrolysis to methylthio-3,5-xylenol, which can be further oxidized to sulfoxide by CYP1A2 and CYP2C19. Carbaryl can be activated via CYP-catalyzed hydroxylation to form more toxic products. Indeed, human CYP1A1 and CYP1A2 have been shown to favor the conversion of carbaryl to 5-hydroxycarbaryl, whereas CYP1A2 and CYP3A4 metabolize carbaryl to 4-hydroxycarbaryl (Figure 2C) [71]. However, chlorpyrifos and cimetidine compete with carbaryl and decrease its metabolism by CYPs. Moreover, both organophosphates and carbamates such as chlorpyrifos, fonofos, carbaryl, and naphthalene influence steroid biosynthesis by inhibiting the 17β-estradiol conversion to 2-hydroxyestradiol by CYP1A2 and CYP3A4 [72]. The inhibition of 17β-estradiol metabolism by chlorpyrifos is irreversible; therefore, this can lead to the estrogen accumulation observed in BC.

### 2.3. Bioactivation vs. Detoxification of Procarcinogens

According to Rendic and Guengerich, human microsomal CYPs catalyze about 66% of all procarcinogen activation pathways, among which >90% were attributed to the following six isoforms: CYP1A1, CYP1A2, CYP1B1, CYP2E1, CYP3A4, and CYP2A6 [73]. In addition to CYPs, SULTs (13%), NATs (7%), AKR (8%), cyclo-oxygenases (2%), and FMOs (1%) are also involved in procarcinogen metabolism. CYP-catalyzed procarcinogen activation occurs, predominantly, due to the reactions of C- and N-hydroxylation, epoxidation, nitroreduction, and conjugation via O-acetylation and O-sulfonation. The products of these bioactivation reactions can interact with macromolecules such as DNA and proteins, causing their damage and cell dysfunction, which is implicated in cancer [74,75].

**Figure 3:**
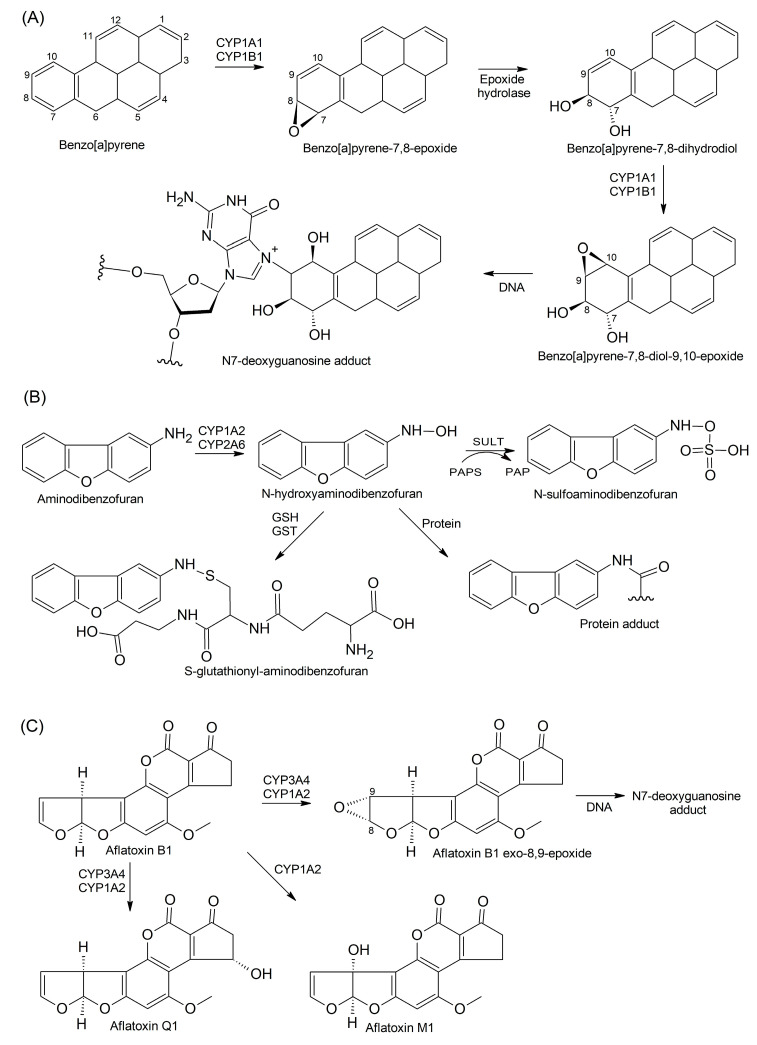
CYP-mediated bioactivation and inactivation of procarcinogens. (**A**) CYP1A1 and CYP1B1 catalyze the conversion of benzo[a]pyrene (B[a]P) to 7,8-epoxy-benzo[a]pyrene, that is further converted by liver epoxide hydrolase to B[a]P-7,8-dihydrodiol, which gives rise to other epoxy-derivatives with the capability to interact with DNA, yielding toxic adducts. (**B**) Metabolic activation of 3-aminodibenzofuran by CYP1A2 and CYP2A6 via the formation of N-hydroxylated derivative, which can either be conjugated with sulfuric acid and GSH or interact with proteins. (**C**) CYP3A4 and CYP1A2 are involved in the inactivation of aflatoxin B1 to its Q1 and M1 derivatives as well as in its activation to exo- and endo-8,9-epoxides that give rise to toxic DNA adducts.

Shimada et al. have investigated the bioactivation of a diverse range of procarcinogens including polycyclic aromatic hydrocarbons (PAHs) such as dibenzopyrenes, chrysene, phenanthrene, and anthracene, as well as their dihydroxy- and dihydro-derivatives [76]. With the use of high-performance liquid chromatographic analysis, the authors showed that CYP1B1 and CYP1A1 catalyze the conversion of procarcinogens into strong carcinogenic metabolites, epoxides. For example, benzo[a]pyrene (B[a]P) yields B[a]P epoxide, which is further reduced to B[a]P-7,8-dihydrodiol in the presence of liver epoxide hydrolase [77]. Subsequently, the B[a]P-7,8-dihydrodiol is converted to other epoxy-derivatives such as B[a]P-7,8-diol-9,10-epoxide, which can interact with DNA to yield toxic adducts (Figure 3A). A kinetic analysis showed that CYP1B1 has 3.2-fold higher V_max_/K_m_ ratio than CYP1A1 for the B[a]P-7,8-diol-9,10-epoxide formation.

Phenanthrene (PH) is not highly toxic to humans compared to B[a]P and 7,12-dimethylbenz[*a*]anthracene (DMBA); however, it has diagnostic value because its metabolites are excreted in higher amounts than other PAHs [78]. Baum et al. have studied conversions of benzo[c]phenanthrene (B[c]PH) to show that microsomal CYP1A2 produces, predominantly, B[c]PH-3,4-dihydrodiol in the liver and B[c]PH-5,6-dihydrodiol in the lung [79]. In genetically engineered V79 Chinese hamster cells, human CYP1A1 and CYP1A2 oxidized B[c]PH at both the 5,6- and 3,4-positions with similar efficiency; however, they displayed regio- and stereoselectivity in B[c]PH activation [80].

Heterocyclic carcinogens, which originate from meat cooked at high temperatures and tobacco smoke, can be activated or inactivated by human liver microsomes and recombinant CYPs. An example is 3-aminodibenzofuran, a carcinogenic aromatic amine with a higher mutagenic activity than B[a]P. It undergoes metabolic activation via N-hydroxylation, and the active metabolite can interact with Cys residues in proteins to form hepatotoxic adducts (Figure 3B). However, the N-hydroxylated metabolite can further conjugate with either sulfuric acid by SULT or GSH by GSTs, and this protects it from interacting with DNA [81]. Another example is dietary-derived coumarin, which itself has hepatotoxic potential [82]; however, it is inactivated via oxidation by CYP1A2 and CYP2A6 to form 2-hydroxyphenylacetic acid and 7-hydroxycoumarin, non-toxic and non-mutagenic metabolites [83].

CYP3A4 and CYP1A2 have been shown to be efficient in metabolizing aflatoxin B1 (AFB1), a carcinogen implicated in the etiology of hepatocellular carcinoma (HCC) [84]. However, CYP3A4 was more active in producing aflatoxin Q1 (AFQ1) and exo-8,9-epoxide, whereas CYP1A2 produced aflatoxin M1, a small amount of AFQ1, and endo- and exo-8,9-epoxides (Figure 3C). AFQ1 and AFM1 formation takes place via hydroxylation, leading to the detoxification of AFB1, whereas endo- and exo-8,9-epoxide formation is an activation reaction. The AFB1 exo-8,9-epoxide is highly reactive and interacts with DNA to form cytotoxic adducts in high yield [85]; however, GSTs catalyze AFB1 exo-8,9-epoxide conjugation with GSH [86].

The major metabolite formed from 3-nitrobenzanthrone (3-NBA), a carcinogen found in diesel exhaust and atmosphere pollution, is 3-aminobenzanthrone (3-ABA). Human and rat liver microsomes and recombinant CYP1A1 and CYP1A2 have been shown to metabolically activate 3-ABA via N-hydroxylation followed by the formation of multiple DNA adducts [87]. Another procarcinogen and industrial pollutant, 2-nitroanisole (2-NA), can be detoxified to 2-nitrophenol (2-NP), which is further oxidized to 2,5-dihydroxynitrobenzene (2,5-DNB) and 2,6-dihydroxynitrobenzene (2,6-DNB) by human CYP2E1, CYP1A1, and CYP2B6 [88]. In rats, 2-NA is activated via nitroreduction by xanthine oxidase to produce *N*-(2-methoxyphenyl)-hydroxylamine and *o*-anisidine; however, the authors have not detected the latter products in humans and rabbits. 2-NP has been shown to be the major metabolite in rabbits and rats, whereas 2,5-DNB is predominantly produced in humans [89].

### 2.4. Metabolism of Drugs

#### 2.4.1. Drugs as CYP Substrates

The liver is a major organ, where drug metabolism takes place; however, CYPs are differently expressed by liver microsomes and vary in their roles in the reactions of drug conversions. CYP3A4, CYP2C9, CYP2C8, CYP2E1, and CYP1A2 are characterized by the highest expression in the liver, whereas CYP2A6, CYP2D6, CYP2B6, CYP2C19, and CYP3A5 are less abundant, and CYP2J2, CYP1A1, and CYP1B1 are mainly expressed in other tissues [90]. Additionally, the CYP-catalyzed metabolism of drugs depends on the activities of CYP redox partners, the involvement of transporters, drug–drug interactions, pathophysiological condition in the liver, etc. [91]. Moreover, at clinically relevant concentrations, only a single or a few CYP isoforms metabolize a drug, even though all CYP isoforms have a broad substrate specificity that overlaps with that of other CYPs.

About three-quarters of all CYP-catalyzed reactions in humans have been attributed to drug metabolism, primarily involving the following five enzymes: CYP1A2, CYP2C9, CYP2C19, CYP2D6, and CYP3A4 [92]. It has been estimated that 27% of reactions of drug metabolism can be assigned to CYP3A4, followed by CYP2D6 (13%), CYP2C9 (10%), CYP1A2 and CYP2C19 (9% each), CYP3A5 (6%), and CYP1A1 (5%). This was confirmed by a meta-analysis undertaken by Achour et al., who have assessed the expression levels of various CYPs to show that CYP3A4 is the most abundant drug-metabolizing CYP in liver microsomes, followed by CYP2E1, CYP2C9, and CYP1A2 [93]. However, results obtained by different research groups can be influenced by variations in measurement methodologies and *CYP* gene polymorphisms, leading to interindividual variability in CYP activities.

**Figure 4:**
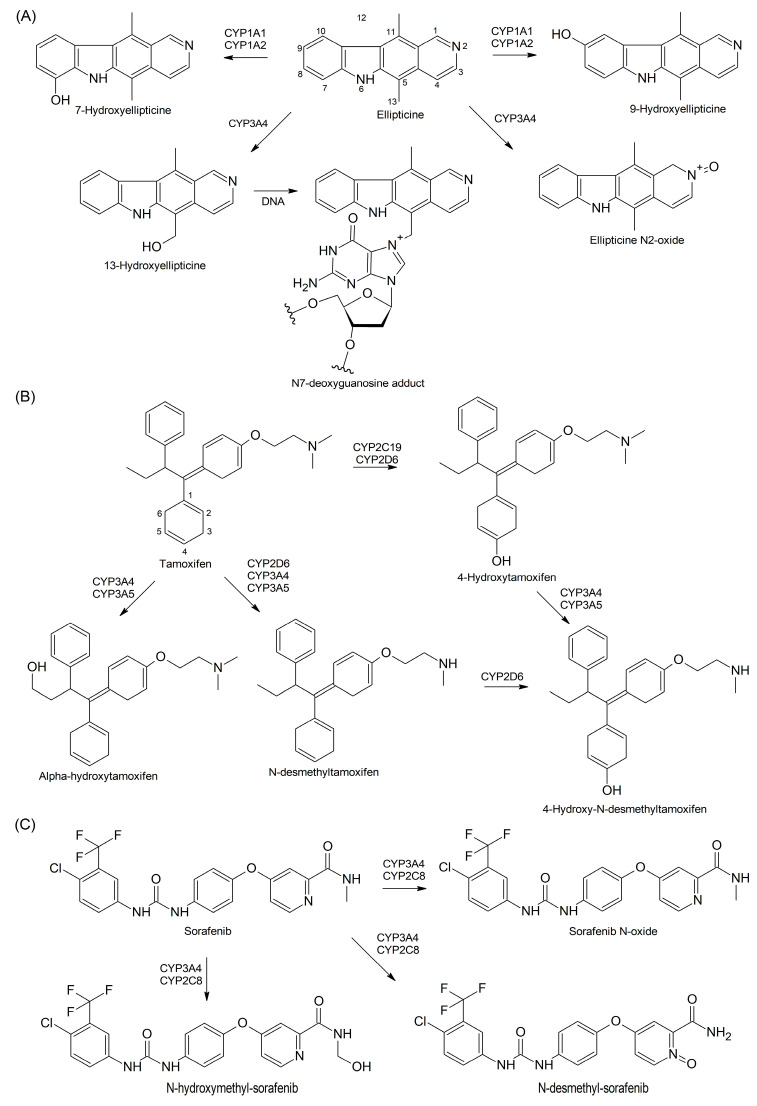
CYP-catalyzed drug metabolism. (**A**) CYP1A1 and CYP1A2 catalyze the ellipticine detoxification by forming 7-hydroxy- and 9-hydroxyellipticine, whereas CYP3A4 catalyzes its bioactivation via its N-hydroxylation to ellipticine N2-oxide and the formation of 13-hydroxyellipticine that can produce DNA adducts. (**B**) Tamoxifen is metabolized to 4-hydroxytamoxifen by CYP2C19 and CYP2D6 and to N-desmethyltamoxifen by CYP2D6, CYP3A4, and CYP3A5. Both metabolites can be converted to 4-hydroxy-N-desmethyltamoxifen by CYP3A4/CYP3A5 and CYP2D6, respectively. CYP3A4/CYP3A5 are also active in converting tamoxifen to α-hydroxytamoxifen. (**C**) Anticancer drug sorafenib is metabolized by CYP3A4 and CYP2C8 to form N-hydroxymethyl, N-desmethyl metabolites, and sorafenib N-oxide.

Various CYPs can metabolize the same substrate to different products, thereby causing either their detoxification or bioactivation. An example is the antitumor drug ellipticine, a CYP substrate and a cell-permeable lipophilic antineoplastic pyrido[3,4-b]carbazole derivative alkaloid that causes DNA intercalation and inhibits topoisomerase II [94,95]. CYP1A1 and CYP1A2 have been shown to catalyze ellipticine detoxification by producing 7-hydroxy- and 9-hydroxyellipticine. On the other hand, CYP3A4 bioactivates ellipticine by generating 12-hydroxy- and 13-hydroxyellipticine and causing its N-hydroxylation to yield ellipticine N2-oxide (Figure 4A) [96]. It has been shown that cyt *b5* increases the efficiency of electron transportation, prompting CYP1A1 and CYP1A2 to produce 12-hydroxy- and 13-hydroxyellipticine. This switch from detoxification to activation causes an increased formation of toxic ellipticine-DNA adducts [97].

Another pyrido[3,4-b]carbazole derivative alkaloid is the antineoplastic drug olivacine (1,5-dimethyl-6*H*-pyrido[4,3-*b*]carbazole), which can be metabolized by CYP1A1, CYP1A2, and FMO3 [98]. Structure–activity relationship analyses have revealed that hydroxylation reactions with the formation of hydroxy-, hydroxymethyl-, and methoxy-derivatives at positions C1, N2, C9, and C11 significantly increase the cytotoxic activity of olivacine toward tumor cells [99]. Indeed, an in vitro study on colorectal cancer cells has shown that the hydroxylation of olivacine at the C9 position with the production of a 9-methoxyderivative increases its cytostatic activity greater than that of ellipticine derivatives and doxorubicin [100].

A semisynthetic analog of the DNA topoisomerase I inhibitor camptothecin (CPT), the anticancer prodrug irinotecan (CPT-11), has been shown to undergo extensive conversions in the liver and intestine, preliminarily by CYP3A4 and CYP3A5 [101]. CYP3A4 has been shown to preferentially inactivate irinotecan via the oxidation of the piperidinylpiperidine side chain, thereby producing 4-N-5-aminopentanoic acid and 4-aminopiperidino derivatives, whereas CYP3A5 catalyzes the de-ethylation of the camptothecin moiety to produce M4 metabolite [102]. Following the treatment with the irinotecan active metabolite produced by carboxylases, 7-ethyl-10-hydroxycamptothecin (SN-38), the pregnane X receptor (PXR) undergoes activation and nuclear translocation. In the nucleus, PXR interacts with the retinoid X receptor (RXR) for heterodimerization and binding to the promoter of genes encoding CYP3A4 and UGT1A1 [103]. This induces gene overexpression, which was most pronounced in primary hepatoma HepG2 cells, whereas the CPT-11 treatment of colon cancer LS180 cells caused their resistance to irinotecan.

Tamoxifen is a selective estrogen receptor-α (ERα) modulator that competitively inhibits the estrogen–receptor interaction, and this provides its usage in the treatment of women with ER-positive (ER+) BC [104]. Tamoxifen is a prodrug that is converted to active metabolites, predominantly, by CYP2D6, CYP3A4, CYP3A5, CYP2B6, and CYP2C19 (Figure 4B). Quantitatively, N-desmethyltamoxifen is a major tamoxifen metabolite constituting 92% of all derivatives and is produced by CYP2D6 and, to a lesser extent, by CYP3A4 and CYP3A5 [105]. Another metabolite, 4-hydroxytamoxifen, is more active and has ~30–100 times more antiestrogenic potency than the parent compound, although this pathway accounts for only 7% of tamoxifen metabolism. CYP2C19’s activity is an order of magnitude higher than CYP2D6 in the formation of 4-hydroxytamoxifen [106].

Both N-desmethyltamoxifen and 4-hydroxytamoxifen can be converted to endoxifen (4-hydroxy-N-desmethyltamoxifen) by CYP2D6 and CYP3A4/CYP3A5, respectively. Endoxifen is the most potent antiestrogen among a variety of other metabolites produced in these pathways and significantly contributes to the therapeutic effects of tamoxifen [107]. The hydroxylation of tamoxifen at the ethyl group causes its conversion to α-hydroxytamoxifen, a genotoxic compound produced predominantly by CYP3A4 in both humans and rats [108]. The toxic adducts of the interaction of α-hydroxytamoxifen with DNA have been identified in the endometrium of women treated with tamoxifen [109]. The production of toxic adducts can be diminished by conjugation with sulfuric acid by SULT1A1 and glucuronic acid by UGTs for further excretion [110]. The CYP-catalyzed metabolism contributes to an intrinsic resistance to tamoxifen fueled by cross-talks with growth factor signaling pathways and hormone-binding transport proteins, which interfere with Erα–hormone interactions [111,112,113].

The molecular mechanisms which underlie tamoxifen resistance may involve *CYP* gene polymorphisms. No statistical differences due to *CYP* gene polymorphisms in the efficacy of tamoxifen have been found by Singh et al. [114]. However, the interindividual differences in postmenopausal women with early BC receiving tamoxifen have been observed by Goetz et al. [115]. The authors have found that patients with decreased tamoxifen metabolism identified as having the CYP2D6*4 genotype exerted significantly shorter recurrence-free survival (RFS) than patients with extensive metabolism. Additionally, Tan et al. have found that five-year RFS and overall survival (OS) were slightly better, but statistically non-significant, in women with the extensive or ultrarapid metabolizer CYP2D6 phenotype compared to those with the intermediate metabolizer phenotype [116]. No significant difference in progression-free survival (PFS) between the CYP2D6*4 genotype group and the overall study cohort has been found by Stingl et al. in the Austrian TIGER study [117].

The stereospecificity of CYP activities in the hydroxylation of the same substrates at the same positions has been observed. Thalidomide, an anticancer drug, is transformed into 5-hydroxythalidomide and the diastereomeric 5′-hydroxythalidomide. In humans, CYP2C19 is the major enzyme catalyzing this reaction, whereas, in rats, CYP2C6 is the predominant isoform hydroxylating thalidomide [118]. Additionally, CYP2A6 and, to a lesser extent, CYP1A2 and CYP2C8 have been shown to convert tegafur, a component of the anticancer drug S-1 used in treating breast, pancreatic, lung, and other carcinomas, to the cytotoxic compound 5-fluorouracil [119]. The conversion takes place via the hydroxylation of the tetrahydrofuran ring at its 5′-position, and the catalytic activity of CYP2A6 is stereospecific [120].

#### 2.4.2. Drugs as CYP Inducers: The Roles of Nuclear Receptors and Efflux Transporters

As mentioned above, anticancer drugs can induce human CYP activity, a process in which human PXR plays a critical role due to its involvement in prolonged drug–drug interactions [121,122]. PXR controls the expression of P-glycoprotein (P-gp) or multi-drug resistance 1 (MDR1) protein, which serves as a transmembrane pump for the ATP-dependent efflux of xenobiotics and drugs [123]. P-gp is a 170 kDa glycoprotein known as ATP-binding cassette subfamily B member 1 (ABCB1) involved in drug resistance and belonging to the superfamily of ABC transporters [124]. Mutations in the *ABCB1* gene encoding the P-gp have been shown to cause the substrate-dependent intracellular accumulation of drugs such as doxorubicin, paclitaxel, and vinblastine, and this may influence patients’ drug sensitivity [125].

The PXR belongs to the superfamily of nuclear receptors (NRs), which function as transcription factors regulating a variety of physiological processes [126]. Despite the high diversity of functions and multiple mechanisms of activation, NRs exhibit a conserved structural organization [127]. Numerous CYP inducers including pregnane, steroid hormones, drugs, and environmental pollutants can activate PXR [128]. For example, PXR is a target for many drugs that induce CYP3A4; it can be activated by high concentrations of flucloxacillin with consequences for the metabolism of other drugs [129]. Flucloxacillin is a β-lactam antibiotic of the isoxazolyl-penicillin group, which can cause liver injury due to the conversion to cytotoxic 5′-hydroxymethylflucloxacillin by CYP3A4 [130]. In addition to CYP3A4, CYP3A7 and CYP2C9 have been implicated in the formation of 5′-hydroxymethyl flucloxacillin and the high variability of hepatic expression of these enzymes may affect the patients’ susceptibility to drug-induced liver injury [131].

Etoposide, a semisynthetic derivative of podophyllotoxin used in BC treatment, undergoes O-demethylation to yield etoposide catechol with the involvement of CYP3A4 and, to a lesser extent, of CYP1A2 and CYP2E1 [132]. Etoposide is pumped by P-gp, and this determines its pharmacokinetics, and, therefore, variation in the transporter expression and activity may influence the oral bioavailability of etoposide in cancer patients [133]. The oral bioavailability of P-gp substrates, in some cases, affects their CYP-catalyzed metabolism. Indeed, in rats with DMBA-induced mammary tumors, the oral administration of etoposide caused an increase in its absorption from the gastrointestinal tract via the inhibition of intestinal P-gp. Additionally, a decrease in the intestinal metabolism of etoposide via the inhibition of both hepatic and intestinal CYP3A has been observed [134].

Hofman et al. have shown that the overexpression of CYP3A4 and CYP2C8 causes a decrease in the sensitivity of HepG2 cells to anticancer drugs from the taxane group such as docetaxel and paclitaxel [135]. CYP3A4-mediated docetaxel resistance due to impaired apoptosis has been observed, and this can be reversed by ketoconazole. CYP3A4 and CYP3A5 catalyze the formation of hydroxy- and dihydroxy-derivatives of paclitaxel and docetaxel, thereby causing their inactivation in cancer cells [136,137]. Consequently, *CYP3A4* and *CYP3A5* gene polymorphisms contribute to interindividual variations in drug clearance and toxicity in BC patients in the PROMIX trial [138]. The frequency of grade 3 and 4 adverse events was higher in the docetaxel poor metabolizer group with the allelic variants CYP3A4*22 and CYP3A5*3.

In cancer cells, PXR ligands enhance PXR-mediated transcription in a ligand- and promoter-dependent manner, thereby leading to differential regulation of CYP3A4 and MDR1. Indeed, in epithelial ovarian cancer (OC) cells, PXR agonists, phthalate and P5, have been shown to induce the expression of CYP3A4 by binding to the *CYP3A4* promoter, whereas paclitaxel and cisplatin increased the MDR1 expression and PXR binding to the *MDR1* promoter [139]. The downregulation of PXR by small interfering RNA (siRNA) inhibited the growth of endometrial cancer (EC) and OC cells and enhanced apoptosis caused by the anticancer drugs, which are also PXR ligands [139,140]. These data suggest that PXR downregulation could be a novel therapeutic approach based on gene silencing to overcome drug resistance in EC and OC treatment.

In addition to PXR, other NRs including the constitutive androstane receptor (CAR) have been shown to recognize drug responsive elements within the 5′’-flanking promoter region of *CYP* genes in response to xenobiotics, steroids, and drugs [141]. Both PXR and CAR were initially considered as orphan receptors with no clear functions; however, they have become recognized as master regulators of xenobiotic metabolism and efflux [142]. CAR has been implicated in the regulation of CYP2B1, CYP2B6, CYP3A4, and CYP2C9 expression in human hepatocytes [143,144,145]. Phenobarbital, rifampicin, and hyperforin have been shown to induce the expression of CYP2C isoforms in primary human hepatocytes, thereby causing increased metabolism of CYP2C substrates in vivo [146]. CAR has been shown to induce the expression of CYP2B6 and CYP3A4 in human primary hepatocytes and leukemia cells, and this enhances the antitumor activity of the alkylating agent cyclophosphamide (CPA) [147]. CPA undergoes bioactivation via conversion to 4-hydroxy-CPA by CYP2C9 and CYP3A4/CYP3A5, a rate-limiting reaction, and to deschloroethyl-CPA, predominantly by CYP3A4 [148].

CAR is involved in the detoxification and clearance of drugs from the liver, and this has clinical relevance [149]. In addition to the liver, CAR is expressed in the epithelial cells of the small intestine, and this expression is required for CYP2B10 and CYP3A11 induction in response to drugs and xenobiotics through the binding of CAR to the *CYP* gene promoters [150]. Additionally, CAR regulates the expression of MDR1 and UGT1A1, thereby affecting the anticancer drug clearance. Wang et al. have observed that effects of anticancer drugs such as cisplatin, paclitaxel, and arsenic trioxide are significantly enhanced by the downregulation of CAR, and this causes the inhibition of OC growth and increased cancer cell apoptosis [151].

#### 2.4.3. CYP Inhibition: Drug–Drug Interactions

In addition to functioning as CYP substrates and inducers, drugs can also serve as CYP inhibitors, among which the most important are those with clinical relevance [152]. Ivermectin serves as both a substrate and inhibitor of human CYPs such as CYP2C9, CYP2C19, CYP2D6, and CYP3A4 and can inhibit drug transporters including P-gp [153]. Ivermectin, a macrocyclic lactone pesticide, is primarily metabolized by CYP3A4 both in vivo and in vitro with some contribution of CYP3A5 and CYP2C9 [154]. 3″-O-demethylivermectin, 4-hydroxymethylivermectin, and 3″-O-demethyl,4-hydroxymethylivermectin have been identified as the major metabolites [155]. Many substrates of CYP3A4, which overlap with those of P-gp, including ivermectin, can inhibit its activity.

CYP2C8 has been shown to increase the anticancer activity of the multi-kinase inhibitor (MKI) sorafenib by converting it to sorafenib N-oxide, thereby inhibiting proliferation, clonality, migration, invasion, and cell cycle progression in cancer cells [156]. Sorafenib is frequently co-administered with other drugs such as paclitaxel for the improvement of the efficacy of anticancer therapy and for the treatment of comorbidities. In this, sorafenib N-oxide, a major pharmacologically active metabolite, demonstrates the potential to contribute to pharmacokinetic drug–drug interactions [157]. Indeed, CYP3A4 forms sorafenib N-oxide, which, along with N-hydroxymethyl and N-desmethyl metabolites (Figure 4C), can inhibit the 6α-hydroxylation of paclitaxel by CYP2C8 [158]. Sorafenib and its metabolites can accumulate in patients’ blood serum during anticancer therapy, and this increases the toxicity of co-administered drugs due to drug–drug interactions [159].

Sorafenib has been shown to suppress the Bcl-2 family member, induced myeloid leukemia cell differentiation protein MCL1, thereby regulating apoptosis in THP-1, U937, and Granta519 cancer cells; however, no increased apoptosis was observed in combination with vinblastine [160]. Vinblastine is metabolized by CYP3A4, and this pathway is inhibited by other anticancer drugs currently co-administered with vinblastine in cancer chemotherapy (etoposide, adriamycin, lomustine, and teniposide) [161]. Another member of the Vinca alkaloid family, vincristine, also inhibits vinblastine metabolism, whereas vincristine itself can be metabolized by CYP3A4 and CYP3A5 through the oxidative cleavage of the piperidine ring of its dihydro-hydroxycatharanthine unit [162].

CYP3A4 has been implicated in the metabolism of another MKI, imatinib, via its N-demethylation to form N-desmethylimatinib, and this correlated with the oxidation of testosterone and midazolam by CYP3A4 and paclitaxel by CYP2C8 [163]. However, during long-term treatment with imatinib, the dose- and time-dependent auto-inactivation of CYP3A4 by imatinib has been observed [164]. Imatinib increased the bleeding risk of rivaroxaban, a widely used direct oral anticoagulant, whereas sunitinib was able to reduce the therapy efficiency due to drug–drug interactions. Imatinib and gefitinib significantly inhibited the CYP2J2- and CYP3A4-mediated metabolism of rivaroxaban as well as the efflux transportation mediated by breast cancer resistance protein (BCRP)- and P-gp [165].

Filppula et al. have shown the ability of a wide range of MKIs to inhibit the activities of CYP3A and CYP2C8. These drugs include bosutinib isomer 1, crizotinib, dasatinib, erlotinib, gefitinib, lestaurtinib, nilotinib, pazopanib, saracatinib, sorafenib, and sunitinib [166]. Among them, bosutinib was the only inhibitor causing the time-dependent inhibition of CYP2C8. The authors also found that midostaurin and nintedanib irreversibly inhibited CYP3A4 and predicted drug–drug interactions between vatalanib and CYP2C8 substrates as well as between masitinib, midostaurin, and vatalanib and CYP3A4 substrates [167]. Despite CYP3A4 and CYP2C8 having been identified as the major contributors to masitinib bioactivation, CYP3A5 and CYP2D6 are also able to form *N*-desmethyl masitinib [168]. Table 1 summarizes the data on the involvement of various human CYP1–CYP4 family members in drug metabolism.

## 3. Roles of CYPs in Cancers

### 3.1. Breast, Endometrial, and Ovarian Cancers

#### 3.1.1. Molecular Mechanisms of Estrogen-Sensitive Cancers

The biosynthesis and metabolism of estrogens play critical roles in the initiation and progression of hormone-sensitive cancers in women—BC, EC, and OC. Among them, BC is the most common cancer type and the fifth leading cause of cancer-related deaths worldwide as estimated in 2020 [1]. BC is a genetically and morphologically highly heterogeneous disease, which is classified based on the immunohistochemical expression of hormone receptors into subtypes: ER+, progesterone-receptor-positive (PR+), human-epidermal-growth-factor-receptor-positive (HER2+), and triple-negative breast cancer (TNBC) with none of the above-mentioned receptors [169]. Approximately 70–75% of invasive BCs are characterized by a significantly high ERα expression, whereas PR is expressed in more than 50% of ER-positive patients [170].

EC is the sixth most common cancer type among women worldwide [171]. EC is also a heterogeneous disease, which is classified, based on the ERα and PR expression, into endometrioid type I (hormone-receptor-positive type) and non-endometroid type II (hormone-receptor-negative type) [172]. The endometrioid type I EC risk has been linked to increased circulating levels of estrogens and persistent ERα activation [173]. OC is the eighth most common cause of death in the female population worldwide and comprises 1.2% of all cancer cases as estimated in 2020 [174]. About 90% of primary malignant ovarian tumors are epithelial carcinomas, and critical roles of hormones in OC development and progression have been reported [175]. For example, studies conducted in the Chinese population have evidenced that ER+ and PR+ statuses were higher among patients with serous and endometrioid carcinomas compared to mucinous and clear-cell carcinomas of malignant epithelial OC, and that ER+ and PR+ OC patients have a better clinical outcome [176].

The current understanding of the molecular mechanisms underlying hormone-sensitive cancers is based on the key roles of CYPs in the biosynthesis and metabolism of steroid hormones, androgens, and estrogens [177]. Recent studies have shown that, in BC, OC, and EC tissues, the levels of circulating estrogens and their metabolites, as well as genotoxic DNA adducts, correlate with the activities of CYPs and conjugating enzymes. Indeed, circulating estrogen levels have been shown to influence ERα activation and DNA adduct formation in BC tissues with the involvement of CYP1B1, SULT1A1, SULT1A2, and GSTP1 [178]. Additionally, the highest expression of CYP1A1 protein has been observed in MCF-7 and MDA-MB-231 BC cell lines and the greatest level of CYP1B1 protein has been reported in SKOV-3 and A2780 OC [179]. However, Leung et al. have found the overexpression of CYP1A1 and no detectable level of CYP1B1 at the transcriptional level in OC cell lines [180].

**Figure 5:**
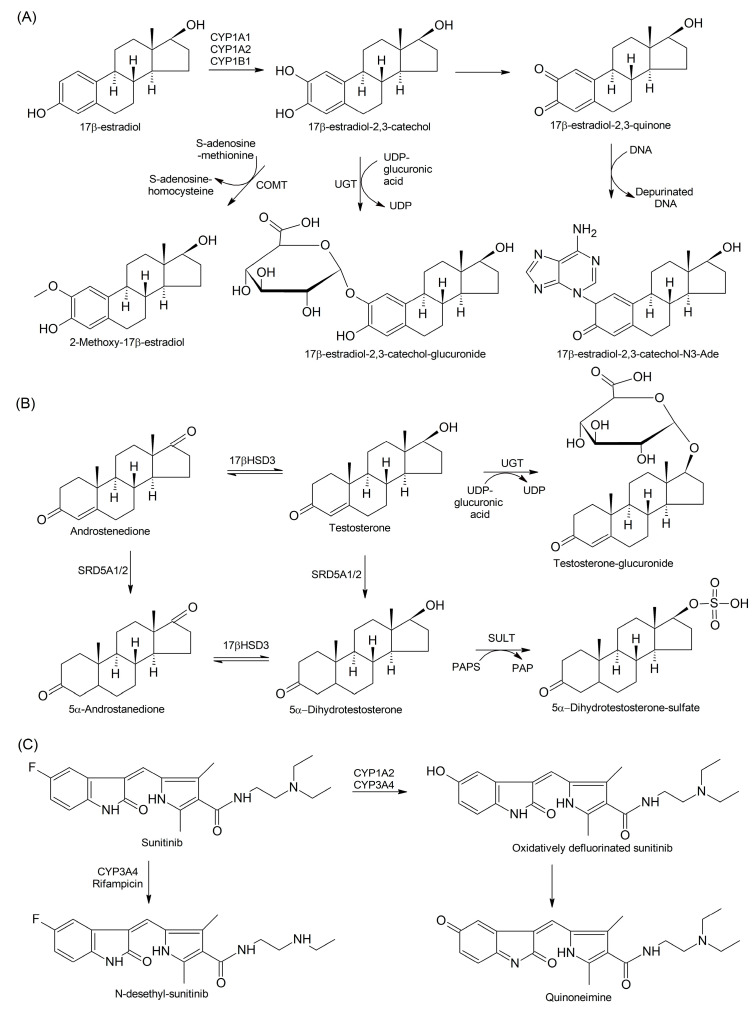
Metabolism of estrogens and androgens by CYPs. (**A**) The conversions of 17β-estradiol by CYP1A1, CYP1A2, and CYP1B1 to 2,3-catechols and, further, to 2,3-quinones can be followed by either methylation by COMT, conjugation with glucuronic acid by UGT, or DNA adduct formation. (**B**) Testosterone can be either reversibly converted to androstenedione by 17βHSD or reduced by SRD5A1/2 isoenzymes to DHT, which can be, alternatively, produced from androstenedione by 17βHSD and SRD5A1/2 isoenzymes. Both testosterone and DHT are mostly conjugated with glucuronic acid and, to a lesser extent, sulfuric acid. (**C**) The metabolism of sunitinib by CYP1A2 and CYP3A4 via oxidative defluorination and the formation of quinoneimine. The presence of the CYP3A4 inducer rifampicin causes sunitinib *N*-dealkylation to form *N*-desethyl-sunitinib.

CYP1A1, CYP1B1, and CYP1A2 are primarily involved in the irreversible hydroxylation of estrone to produce catechol derivatives, 2-hydroxy- and 4-hydroxy-estrone, whereas CYP2C19 is mostly responsible for the formation of 16α-hydroxy-estrone [181]. The 2-hydroxylation and 4-hydroxylation of 17β-estradiol have been shown to correlate with the mono-oxygenase activities of CYP3A4 and CYP3A5 in human liver microsomes [182]. Estrogens undergo 2-hydroxylation to yield 2,3-catechols, whereas their 4-hydroxylation gives rise to 3,4-catechols. Both types of catechol are further converted to corresponding 2,3- and 3.4-quinones involved in the formation of toxic protein and DNA adducts (Figure 5A). Mutagenic adduct formation with adenine and guanine causes DNA depurination, and this can serve as a biomarker for estrogen metabolite formation in BC [183].

The ability of hydroxy-metabolites of estrogens to cause BC cell proliferation, and, therefore, their tumorigenic activity, depends on the position of the hydroxylated atom. Indeed, 16α-hydroxy-metabolites of 17β-estradiol and estrone have been found to exhibit ERα agonist properties and mitogenic activity in ER+ MCF-7 and T47D human BC cells [184]. On the contrary, 2-hydroxy-metabolites of both 17β-estradiol and estrone have shown weak mitogenic activity and no ERα agonist properties in the both cell lines, instead displaying protective effects. Moreover, 17β-estradiol and its metabolite, 4-hydroxyestradiol, have been reported to protect from oxidative stress via increasing superoxide dismutase (SOD) activity, whereas 16α-hydroxy-estrone increased GST activity [185].

The tumor-protective activities of estrogen hydroxy-metabolites have been attributed to the formation of methoxy-derivatives and conjugated estrogens [186,187]. Indeed, catechol estrogens can be methylated by catechol-O-methyltransferase (COMT) or conjugated, for example, with glucuronic acid by UGTs [188]. The O-methylation of catechol estrogens by COMT can serve as a protective pathway via the minimization of depurinating DNA adduct formation and cancer prevention [189,190]. A case–control study of serum levels of 15 estrogens and their metabolites in both conjugated and unconjugated forms has shown that almost all estrogens and estrogen metabolites in the unconjugated form are associated with an increased BC risk in postmenopausal women [191]. Among O-methylated metabolites, methoxy-derivatives of 3,4-catechols were genotoxic and associated with a higher BC risk, whereas non-methylated 2-hydroxy-derivatives of estrogens were associated with a lower risk in postmenopausal women.

The inhibition of COMT has been shown to decrease the formation of the 2-methoxy-derivative from 2-hydroxy-17β-estradiol, and this increases the oxidative DNA damage via the 8-hydroxy-2′-deoxyguanosine (8-oxo-dG) formation in MCF-7 cells exposed to 17β-estradiol [192]. Additionally, increased levels of albumin and hemoglobin protein adducts formed by 2,3- and 3,4-quinones of 17β-estradiol have been shown to correlate with worse 5-year survival in BC patients [193]. The success of BC treatment with the use of aromatase inhibitors and tamoxifen can be associated with a dramatic reduction in the estrogen quinone burden.

The increased levels of unconjugated 17β-estradiol, estriol, and estrone and their metabolites have been associated with a higher cancer risk in women with type I as compared to type II EC [173]. Despite the fact that circulating progesterone levels were not correlated with either OC or EC, the increased progesterone-to-estradiol ratio was negatively associated with EC risk [194]. Additionally, the increased level of 17α-OHP5 was negatively associated with EC risk and positively associate with OC risk. Women with OC had a higher urinary depurinating DNA adduct/estrogen ratio compared to healthy women, and this has been associated with the presence of two low-activity alleles of the *COMT* and one or two high-activity alleles of the *CYP1B1* gene [195].

#### 3.1.2. *CYP* Gene Polymorphisms in Estrogen-Sensitive Cancers

Various SNPs in genes encoding CYPs involved in estrogen biosynthesis and metabolism have been implicated in an increased risk of BC. Indeed, SNPs in the *CYP1A1* gene have been identified to positively correlate with ER+ BC in different populations. Martínez-Ramírez et al. have found that polymorphisms in genes encoding ERα, CYP1A1, CYP1B1, and COMT, are positively associated with BC risk in Mexican women [196]. However, no statistically significant correlation between SNPs in genes encoding CYP1A1 and the north Indian population has been found [197]. Additionally, the heterozygous *CYP1A1* TC genotype was associated with a reduced BC risk in Nigerian women, whereas the homozygous CC genotypes had no association [198].

Zhang et al. have found that BC risk in postmenopausal Chinese women is associated with the *CYP17* TC genotype, unlike the TT genotype [199]. The authors also found that the *CYP19* TC+TT genotypes were associated with both overall cancer risk and premenopausal cancer risk, particularly for ER+/PR+ tumors. In a multigenic study, the *SULT1A1* His allele has been shown to be the most significant BC determinant because its frequency was significantly higher than in controls, associating with the presence of lymph node metastasis in the Chinese population [200]. The *CYP19* (TTTA)_10_ allele was associated with tumor size, whereas the frequency of the *CYP17* A2 allele did not differ from controls. In addition to *CYP19* alleles containing 7 to 13 TTTA repeats identified in many populations, a novel (TTTA)_6_ allele and a significant positive association between the (TTTA)_10_ allele and BC have been described in the Brazilian population [201]. The (TTTA)_10_ allele was three times more frequent with a significant positive association with BC. A combined analysis of two haplotypes, *CYP17* A1 and *CYP19* TT, has suggested that they play protective roles in BC patients [202].

A case–control study of the associations between the *CYP1B1* and *COMT* polymorphisms and invasive EC risk revealed that carriers of the *CYP1B1* N453S allele had a statistically significant decrease in cancer risk. However, in the same study, no significant association was found between the *CYP1B1* L432V allele and EC risk [203]. The associations of *CYP1B1* R48G (142C > C) and *ERα* 975C > G polymorphisms with EC cancer risk have been identified in the Polish subpopulation of Caucasians [204]. In the *CYP1A1* gene, three genotypes (Msp1, I462V, and T461N) have shown no additional risk of EC in Caucasian women [205]. Nevertheless, associations between the rs2470893 polymorphism of the *CYP1A1* gene and EC and OC in Mediterranean women have been identified [206].

No significant association between L432V polymorphism in the *CYP1B1* gene and OC risk has been found [207]. Additionally, no association between the *CYP1B1* rs1056827 polymorphism and OC risk have been identified in a meta-analysis, despite the rs1056836 polymorphism was associated with OC risk in chemotherapy-sensitive and drug-resistant patients among Caucasian and Asian populations [208]. However, in the same study, no significant associations were found between the rs1056836 polymorphism and chemotherapy-resistant patients among African-Americans. 

### 3.2. Prostate Cancer

#### 3.2.1. Molecular Mechanisms of Prostate Cancer

PCa is the fourth most common cancer worldwide and the eighth leading cause of cancer-related deaths worldwide as estimated in 2020 [209]. Many early-stage PCa cases are androgen-dependent or castration-sensitive; therefore, androgen production, metabolism, and transport greatly influence PCa progression [210]. Consequently, decreasing androgen levels in the body or blocking their action may be an effective method of the anticancer therapy [211].

CYP17A1 is a single enzyme that catalyzes the sequential 17α-hydroxylase and 17,20-lyase steps of the conversion of P5 and P4 to DHEA and androstenedione, androgen and estrogen precursors [212]. In humans, the *CYP17A1* gene is expressed in the gonads, adrenal glands, and prostate cancer cells, and both 17α-hydroxylase and 17,20-lyase activities are required for sex steroid hormone biosynthesis [213]. During human development, CYP17A1 is found in the ER of testicular Leydig cells and the adrenal cortex, the major sites of testosterone biosynthesis. Additionally, in cells with high 17,20-lyase activity, POR and cyt *b*_5_ become present [214].

With the use of an in vivo LNCaP PCa mouse xenograft model, the circulating cholesterol level has been shown to significantly correlate with tumor size and intra-tumoral testosterone and CYP17A levels [215]. Moreover, a correlation between the expression of CYP17A1 and the nuclear androgen receptor (AR) at both the protein and mRNA levels has been observed in 50% of the hormone-dependent 22Rv1 cell line [216]. Consistent with these data, the expression of CYP17A1 has been demonstrated in 91% of benign prostate hyperplasia (BPH) specimens and 83% of PCa specimens [217]. Additionally, CYP11A1, responsible for pregnenolone production and the translocator protein (TSPO), which transports cholesterol into mitochondria, have been expressed in all BPH and PCa specimens.

For advanced PCa, androgen deprivation therapy (ADT) by surgical or medical castration has been recognized as a standard treatment. Initially, ADT is effective in most patients; however, it can lead to the development of uncurable metastatic castration-resistant prostate cancer (CRPC) [218]. The progression of ADT-responsive PCa to CRPC can be explained by the overexpression and mutations in the *AR* gene. However, metastatic CRPC has been shown to exist as a mixture of cells displaying varying AR expression levels [219]. The sustained intra-tumoral synthesis of 5α-dihydrotestosterone (DHT), a more potent AR agonist than testosterone, also contributes to CRPC [220,221]. Testosterone can be either reversibly converted to androstenedione by 17βHSD or reduced by SRD5A1/2 isoenzymes to DHT (Figure 5B). Alternatively, DHT can be produced from androstenedione via its sequential conversions to 5α-androstanedione (5α-dione) by SRD5A1/2 isoenzymes and to DHT by 17βHSD.

Since persistent androgen signaling has been implicated in CRPC progression and metastasis, studies are being conducted to develop AR antagonists [222]. However, PCa cells can develop mechanisms to resist the AR blockade due to overexpression, mutations, and alterations in coregulators of AR. Therefore, some studies have attempted to develop various potential steroidal and non-steroidal derivatives with the capabilities of reversible small-molecule CYP inhibitors [223,224]. Metastatic CRPC has been shown to display the upregulation of genes encoding steroidogenic CYP enzymes including CYP17A1 and CYP19A1 [225]. Therefore, various potential steroidal and non-steroidal CYP17A1 inhibitors have been proposed as candidates for CRPC treatment [226,227,228,229,230,231]. However, the only FDA-approved drug for metastatic CRPC treatment is the steroidal irreversible selective CYP17A1 inhibitor abiraterone [232]. Abiraterone acetate, an orally administered small molecule derived from the structure of P5, has been shown to selectively and irreversibly inhibit both the 17α-hydroxylase and 17,20-lyase activities of CYP17A [233]. This activity is ~10–30 times greater than that of the non-selective inhibitor of CYP17A1 ketoconazole.

In a randomized phase 3 clinical trial (NCT00638690), 1195 patients with metastatic CRPC who had previously received docetaxel were assigned to a combined treatment with prednisone and abiraterone acetate and showed an increase in OS (14.8 months) as compared to the placebo group (10.9 months) [234]. Another clinical trial involving 57 patients with metastatic CRPC has evidenced that abiraterone acetate causes a reduction in testosterone level in both the blood circulation and bone marrow [235]. Limitations in the use of abiraterone have been found due to its steroidal structure and the ability to inhibit other pathways, in which AR is involved as a transcriptional regulator.

Recent advancements in bioinformatics and computational approaches have enabled the discovery of novel CYP17A1 inhibitors to replace abiraterone and elucidating the mechanisms underlying CYP–inhibitor interactions. For example, CYP17A1 inhibitors, seviteronel and abiraterone, have been demonstrated to function as competitive AR antagonists [236]. A molecular dynamics (MD) simulation study has revealed the importance of conserved H-bonds for acquiring the proper position by abiraterone in the CYP17A1 binding site [237]. MD simulation has also demonstrated the extended residence time of abiraterone within the CYP17A1 active site, even after abiraterone has been mostly eliminated [238]. This has been experimentally confirmed by the prolonged suppression of DHEA observed in VCaP cells after the abiraterone washout. The authors concluded that their findings can provide a basis for re-evaluating the current dosing regimen in abiraterone treatment while simultaneously minimizing adverse events.

Additionally, studies have been undertaken to discover novel molecular targets in PCa treatment. For example, Zhang et al. have shown that the genetic or pharmacological activation of PXR decreases androgenic activity by inducing the expression of CYP3A4 and SULT2A1 [239]. Treatment with rifampicin, the PXR agonist, inhibited the androgen-dependent proliferation of LAPC-4 cells, but had little effect on the growth of the androgen-independent isogenic LA99 cells. PXR is expressed at the mRNA and protein level in both rat primary Leydig cells and mouse Leydig tumor MA-10 cells, and the incubation of these cells with pregnenolone 16α-carbonitrile resulted in a significant decrease in testosterone biosynthesis [240]. This was associated with the decreased protein expression of key steroidogenic enzymes such as CYP17A1 and 3βHSD.

#### 3.2.2. *CYP* Gene Polymorphisms in Prostate Cancer

Polymorphisms in genes involved in the testosterone biosynthetic pathways can strongly affect the progression of androgen-dependent cancers. Among the polymorphisms playing roles in PCa etiology, those in genes encoding AR, the prostate-specific antigen (PSA), SRD5A2, CYP17A1, CYP3A4, and a putative hereditary PC susceptibility 2 protein (HPC2/ELAC2) have been suggested [241]. For example, the *HPC2/ELAC2* has been found to feature the S217L and A541T polymorphisms, whereas the *SRD5A2* features the A49T and V89L polymorphisms. A statistically significant correlation between the A49T mutation in the *SRD5A2* gene and BPH in Turkish men has been reported [242]. Additionally, the *SRD5A2* V89L variant has been suggested to influence the risk of PCa among younger-aged men with a diagnosis of more aggressive disease [243].

Some studies have shown that there is no evidence of associations between the *CYP17* and *SRD5A2* polymorphisms and PCa risk [244,245]. However, Lindström et al. have found that PCa risk is significantly associated with multiple SNPs in the *AR* and *CYP17* genes, as well as with one SNP in the *SRD5A2* gene [246]. Mutations in individual genes encoding ERα and CYP17 increased the PCa risk by 2 and 3.5 times compared to BPH and healthy controls, respectively. However, a combination of mutant alleles of the two genes increased the cancer risk by more than 2 times among the North Indian population [247]. Another study has found no significant association between the *SRD5A2* gene polymorphism and PCa risk, but has identified a significant association between the *CYP17* gene polymorphism and PCa risk in the Japanese population [248].

Additionally, a case–control study of genes encoding CYP1A1, CYP1A2, CYP2E1, GSTM1, and GSTT1 has shown that only a combination of polymorphisms in *CYP1A1* and *GSTM1* is associated with PCa risk in the Japanese population [249]. The increased risk of PCa in the Japanese population was observed among individuals with the phase II enzyme *N*-acetyltransferase 2 (NAT2) slow acetylator (NAT2*5, NAT2*6, and NAT2*7) genotypes, as well as the *CYP1A1* GG, *CYP1A1* GA+GG, *CYP1A2* CA, and *CYP1A2* CA+AA genotypes [250]. In the latter study, the *CYP1A1* GA+GG genotype was associated with the intake of heterocyclic aromatic amines, which are CYP substrates. 

Polymorphisms in the *CYP1B1* gene have also been studied to show that combinations of some of them are associated with an increased PCa risk, whereas one frequent haplotype (TATGT) is associated with a decreased cancer risk [251]. Some *CYP1B1* haplotypes were positively associated with PCa among men with a highly aggressive disease [252]. A meta-analysis conducted on various cancer types has shown that the A119S polymorphism in the *CYP1B1* gene is associated with PCa risk among Caucasians [253]. Such genetic variants within the *CYP1B1* gene have been suggested to increase the susceptibility to PCa by altering the telomere length [254]. Importantly, inherited genetic variations in the *CYP1B1* gene can serve as predictors of clinical outcomes in patients with clinically localized PCa [255].

In a meta-analysis conducted for the Asian population, the *CYP1B1* gene rs1048943 and rs4646903 polymorphisms were associated with a significant increase in PCa risk. However, no association has been identified between the rs10012, rs162549, rs1800440, and rs2551188 polymorphisms and PCa risk [256]. A significant association between the rs1048943 polymorphism in the *CYP1A1* gene and PCa risk has been found in the overall population, but not in Caucasians. Another case–control study revealed no significant association between PCa risk and the *CYP1A2* rs2472299 and rs11072508 polymorphisms [257].

The CYP3A4*1B haplotype has been found to be positively associated with PCa risk in Caucasians with a more aggressive disease, whereas the CYP3A4*1B/CYP3A5*1 haplotypes were negatively associated with PCa risk in Caucasians with a less aggressive disease [258]. Data obtained by a meta-analysis conducted by Liang et al. have shown that the CYP3A5*3 gene polymorphisms may associate with an increased PCa risk, particularly in African populations [259]. Bioinformatics tools have enabled the identification of a significant association between the CYP3A4*1B and CYP3A5*3 haplotypes and PCa risk and disease progression [260]. The combination of the CYP3A5*3/*3 and *SRD5A2* A49T GG genotypes was associated with tumor clinical stages T2–T4, whereas the CYP3A5*3/*3 and *KLK3* I179T CC/TC genotypes had increased OS in patients with metastatic PCa [261]. Additionally, family-based analyses have made it possible to identify an association between PCa risk or aggressiveness and several *CYP3A4* SNPs and two *SRD5A2* SNPs [262].

### 3.3. CYPs, Hepatotoxicity, and Liver Cancer

#### 3.3.1. Anticancer-Drug-Induced Liver Injury

The expression and activities of CYP enzymes have been shown to change during liver injury including in chronic alcoholic liver disease and nonalcoholic fatty liver disease (NAFLD) [263]. In a mouse model of acetaminophen (APAP)-induced liver injury, a decreased expression and activities of CYP3A11, CYP1A2, CYP2B10, CYP2C29, and CYP2E1 have been observed [264]. In this, adult male mice were more susceptible to APAP-induced hepatotoxicity and showed a greater decrease in the expression of CYPs compared to female mice. It has been shown that the expression and activity of CYP1A, CYP2C19, and CYP3A change significantly during liver diseases, whereas CYP2D6, CYP2C9, and CYP2E1 are less affected [265].

On the other hand, it is well-documented that CYP-activated drugs and procarcinogens produce metabolites, which induce hepatotoxicity. For example, the induction of hepatic cytochrome CYP2E1 catalyzes the conversion of APAP to the hepatotoxic metabolite, N-acetyl-p-benzoquinone imine (NAPQI) [266]. APAP-induced hepatotoxicity can be potentiated by isoniazid and ethanol, which also induce CYP2E1, thereby increasing by 2 to 3 times the formation of NAPQI [267]. Additionally, 8-methoxypsoralen (8-MOP) significantly reduced APAP-induced hepatotoxicity due to the inhibition of CYP2E1 enzymatic activity in mice [268]. The 8-MOP binds to the CYP2E1 active site, thereby competitively inhibiting the oxidative metabolism of APAP. The expression and activity of CYP2E1 can be inhibited by another natural chemical, silymarin, as shown with the use of human and rat liver microsomes and HepG2 cells [269].

Sunitinib, an MKI inhibitor, undergoes metabolic activation via aromatic and aliphatic hydroxylation, N-oxidation, and oxidative defluorination [270]. Sunitinib malate is an orally administered drug approved for the treatment of metastatic tumors; however, its effectiveness is limited in certain cancers such as BC [271]. Moreover, CYP1A2 and CYP3A4 and, to a lesser extent, CYP2D6 catalyze the oxidative defluorination of sunitinib, followed by its bioactivation to quinoneimine (Figure 5C), which causes idiosyncratic hepatotoxicity. However, the quinoneimine can interact with GSH to form a quinoneimine-GSH conjugate, and this decreases the hepatotoxicity of sunitinib [272]. In human hepatocytes, the CYP3A4 inducer rifampicin significantly increases the sunitinib N-dealkylation to the primarily active metabolite N-desethyl-sunitinib [273].

The metabolic activation of the dual-tyrosine kinase inhibitor lapatinib by CYPs has also been implicated in idiosyncratic hepatotoxicity [274]. The following three primary metabolites of lapatinib, O-dealkylated lapatinib, N-dealkylated lapatinib, and N-hydroxy-lapatinib, are produced in HepG2 cells, which overexpress CYP1A1, CYP3A4, CYP3A5, and CYP3A7 [275]. The N-dealkylated lapatinib has been shown to be the most toxic metabolite, which causes DNA damage and cell apoptosis. However, CYP3A4 and CYP3A5 catalyze the O-dealkylation of lapatinib, followed by the formation of the quinonimine-GSH adduct [276].

One of the mechanisms underlying the hepatotoxic effects of anticancer drugs is their photobiological properties, which contribute to CYP-induced photo(geno)toxicity as shown under in vitro conditions. Indeed, both lapatinib and its active N-dealkylated derivative produced by CYP3A4 and CYP3A5 have been shown to exert photo- and genotoxicity, which cause the photosensitized protein and DNA damage [277]. Another anticancer drug, gefitinib, a selective epidermal growth factor receptor inhibitor, undergoes metabolic bioactivation by CYP3A4, CYP3A5, CYP1A1, and CYP2D6 [278,279]. This results in the formation of O-demethyl-, 4-defluoro-4-hydroxy-, and O-demorpholinopropyl-derivatives. Among these, the O-demethyl gefitinib has the highest photo(geno)toxicity and causes DNA damage [280].

#### 3.3.2. Liver Cancer and *CYP* Gene Polymorphisms

HCC is the sixth most common primary malignant tumor of the liver with an increasing incidence rate worldwide and the third most frequent cause of cancer-related deaths worldwide as estimated in 2020 [281]. Liver cirrhosis and inflammation associated with hepatitis B virus (HBV) or hepatitis C virus (HCV) are the main causes of HCC [282]. The exposure to the procarcinogen and genotoxic agent AFB1 is a crucial factor in promoting HCC in individuals infected with HBV [283]. AFB1 has been shown to induce CYP expression by triggering the nuclear translocation of the aryl hydrocarbon receptor (AHR), which functions as a transcription factor capable of binding to *CYP* gene promoters [284].

Silvestri et al. have studied polymorphisms in genes encoding CYP1A1, CYP1A2, CYP2D6, CYP2E1, and CYP3A4 as risk factors for liver cancer progression in HCV-infected patients [285]. They found that the *CYP2D6* poor metabolizer genotype was significantly less frequent in hepatitis/cirrhosis and HCC patients compared to healthy subjects. The presence of two risk alleles in the *CYP2D6* gene in the same rapid-metabolizer subjects demonstrated an increased risk of HCC [286]. In the same study, an association between the *NAT2* slow acetylator genotypes and HCC risk was found in patients who lacked serum HBV and HCV biomarkers. In HCC patients with the homozygous poor metabolizer CYP3A5*3 and CYP3A5*1 genotypes, the CYP3A5 protein has not been found [287]. Moreover, CYP3A5 has been suggested to serve as a tumor suppressor since lower CYP3A5 levels were associated with a more aggressive disease characterized by vascular invasion, poor differentiation, and shorter RFS and OS in HCC patients [288]. Therefore, inhibiting CYPs to overcome drug resistance requires taking into account the variability in *CYP* gene polymorphisms among different ethnic groups [289].

The CYP2D6*10 mutant homozygous genotype has been shown to be 2.8 times less frequent in HCC patients than in controls, whereas the CYP2C9*3 and CYP3A5*3 polymorphisms have been shown to cause changes in enzyme activity [290]. A statistically significant difference in genetic mutant alleles has been identified for the CYP2A6*4A homozygous genotype in HCC patients, which was significantly higher than that in healthy subjects of the Japanese population [291]. The CYP2D6*10 (TT) genotype has also been shown to be associated with a decreased risk of HCC due to the decreased activity of the enzyme [292].

The *CYP24A1* rs6013897 genotype has been shown to be associated with cirrhosis and HCC [293], whereas the c1/c1 genotype of the *CYP2E1* gene was higher in patients with HCC than in controls [294]. The presence of any of the CYP2E1*5B and CYP2E1*6 variant alleles were inversely associated with the HCC risk [295]. There was a borderline increased risk among individuals who carried both the CYP1A1*2A and *SULT1A1* variant alleles. A significant association was observed between the *GSTT1* gene expression level and smoking, and a borderline significant association was observed between the *SULT1A1* gene expression level and smoking.

Diethylnitrosamine induces liver injury and hepatocarcinogenesis due to the metabolic activation by CYP2E1, which strongly correlates with the incidence and severity of liver cancer [296]. The lower activity of CYP2E1 accompanied by the *POR* rs10954732 (G>A) polymorphism has been shown to decrease the susceptibility to HCC, leading to a 199% increase in OS [297]. POR is downregulated in HCC cells and can be upregulated by knocking out the gene that encodes glucose-6-phosphate-dehydrogenase (G6PD) [298]. G6PD suppresses ferroptosis, increases cancer cell viability, and promotes metastasis, which are associated with poor OS in HCC patients. Table 2 summarizes the findings on the statistically significant association between CYP polymorphisms and cancer risk discussed here.

## 4. Conclusions and Challenges

Human CYP enzymes demonstrate broad and overlapping substrate specificities with a balance between enzyme activities and substrate preferences that vary depending on a variety of factors. These factors include CYP isoforms, *CYP* gene polymorphisms, tissue type, developmental stage, age, sex, and ethnicity. Indeed, CYP17A1 possesses both 17α-hydroxylase and C17,20-lyase activities, playing a role in the conversion of several substrates in steroid biosynthesis pathways. CYP17A1 is required for converting P5 and P4 to DHEA and androstenedione, which are androgen and estrogen precursors. Moreover, the same CYP isoform can catalyze both bioactivation and detoxification reactions depending on the substrate and type of reaction. For example, human CYP1A1 activates a diverse range of procarcinogens to produce strong carcinogenic metabolites, epoxides. Additionally, CYP1A1 has been shown to catalyze the activation of carbaryl to the more toxic 5-hydroxycarbaryl; however, it catalyzes the detoxification of 2-nitoanisole to the O-demethylated metabolite, 2-nitrophenol. Furthermore, the same CYP isoform can convert the same substrate to different products depending on the tissue type. For example, microsomal CYP1A2 predominantly converts B[c]PH to B[c]PH-3,4-dihydrodiol in the human liver and B[c]PH-5,6-dihydrodiol in the lung.

On the contrary, different CYP isoforms can convert the same substrate to a variety of products, which are either more toxic or less toxic than their parent compound. Indeed, CYP2B6 is a main CYP enzyme responsible for the bioactivation of chlorpyrifos with the formation of more toxic oxon derivatives, whereas CYP2C19 is primarily responsible for chlorpyrifos detoxification to TCP. Additionally, the CYP3A4-catalyzed metabolism of AFB1 causes its conversion to the less toxic AFQ1, whereas CYP1A2 converts it to the more toxic exo- and endo-8,9-epoxides. Another example is the antitumor drug ellipticine, which is detoxified by CYP1A1 and CYP1A2 via the formation of 7-hydroxy- and 9-hydroxyellipticine. However, CYP3A4 produces 13-hydroxyellipticine via the reaction of N-hydroxylation to yield ellipticine N2-oxide, the metabolites responsible for the formation of toxic DNA adducts. Tamoxifen, another anticancer drug, is metabolized to 4-hydroxytamoxifen by CYP2C19, whereas CYP3A4 and CYP3A5 convert tamoxifen to N-desmethyltamoxifen, further metabolized to endoxifen by CYP2D6. All these conversions enhance the therapeutic efficiency of tamoxifen; however, the hydroxylation of tamoxifen at the ethyl group by CYP3A4 causes its conversion to α-hydroxytamoxifen, a genotoxic compound that produces DNA adducts.

The involvement of CYPs in drug metabolism underlies drug–drug-interactions and drug resistance. These processes are under the control of nuclear receptors such as PXR, CAR, and VDR that regulate the expression of P-gp (MDR1), which serves as a transmembrane pump for drug efflux. PXR and CAR can be activated by numerous CYP inducers including pregnane, steroid hormones, drugs, and environmental pollutants. Various PXR and CAR ligands and anticancer drugs including cisplatin, docetaxel, paclitaxel, and arsenic trioxide cause the upregulation of P-gp. These nuclear receptors bind to the promoter of *CYP* genes to induce their transcription in response to xenobiotics and drugs. Anticancer drugs and their metabolites can be accumulated in the blood serum of patients during therapy, which may increase the toxicity of co-administered drugs due to drug–drug interactions. For example, imatinib can increase the bleeding risk of rivaroxaban, a widely used direct oral anticoagulant. Imatinib and gefitinib significantly inhibit the CYP2J2- and CYP3A4-mediated metabolism of rivaroxaban and its efflux transportation mediated by the breast cancer resistance protein (BCRP)- and P-gp.

CYP-catalyzed steroid hormone production and metabolism play critical roles in the development and pathogenesis of hormone-sensitive cancers in men and women—prostate, breast, endometrial, and ovarian cancers. The current understanding of the molecular mechanisms underlying hormone-sensitive tumors is based on dysregulated CYP expression and mutagenic adduct formation, which cause the cellular accumulation of toxic protein and DNA adducts. CYP1A1, CYP1A2, CYP1B1, and CYP3A4/CYP3A5 are the most important CYPs involved in the irreversible hydroxylation of estrogens to produce catechol estrogens, whereas CYP2C19, mostly, forms the 16α-hydroxy-derivative. However, the 16α-hydroxy-derivatives of estrogens serve as ERα agonists, whereas their 2-hydroxy- and 4-hydroxy-derivatives along with methoxy-derivatives and conjugated estrogens exhibit tumor protective activities. In prostate cancer, key roles have been attributed to CYP17A1 and the sustained intra-tumoral synthesis of 5α-dihydrotestosterone, which is a more potent AR agonist than testosterone. This dictates the importance of anticancer drug development based on steroidal and non-steroidal CYP and hormone receptor inhibitors.

The variations in CYP activities are provided by diverse factors including *CYP* gene polymorphisms, which affect interindividual differences in cancer and drug susceptibilities. Various SNPs in genes encoding CYPs involved in estrogen and androgen biosynthesis and metabolism have been implicated in the increased risk of hormone-sensitive cancers. However, polymorphisms in only some CYP isoforms have been identified to show statistically significant associations with breast, endometrial, ovarian, prostate, and liver cancers in different ethnic groups. Therefore, more data are needed to clearly elucidate the associations between different types of *CYP* gene polymorphisms and cancer susceptibility in various populations. Currently, data on such associations are limited to a few polymorphisms and a small number of populations.

Additionally, many questions remain regarding the involvement of CYPs in drug metabolism and adverse drug reactions, which underlie the patients’ susceptibility to treatment. The steroidal nature of CYP inhibitors and their ability to inhibit other pathways, including those involving hormone receptors as transcriptional regulators, are also challenging. In this context, silencing the *CYP* genes by small RNAs or CYP activation could be a strategy to overcome the resistance to drugs, which serve as CYP substrates, inducers, and inhibitors. Understanding how CYP-catalyzed drug metabolism impacts drug–drug interactions and drug resistance may provide a strategy for personalized chemotherapy in specific patient groups. Data on how *CYP* polymorphisms determine drug metabolizer phenotypes, disease outcomes, and patients’ survival could further guide treatment decisions. However, the effectiveness in CYP inhibiting CYPs to overcome drug resistance may vary due to the differences in *CYP* gene polymorphisms among different ethnic groups, necessitating further research in this field. Since polymorphisms and mutations in genes encoding CYPs and other drug-metabolizing enzymes as well as receptors are involved in cancer and drug susceptibility, high-throughput genomic technologies could be beneficial for cancer patients.

## Figures and Tables

**Table 1:** Human CYP-catalyzed anticancer drug metabolism.

CYP Type	Name of a Drug	Action of the CYP	Product of the CYP Action	References
CYP1A1	Ellipticine	Detoxification	7-hydroxyellipticine, 9-hydroxyellipticine	[96]
	Olivacine	Activation	hydroxy-, hydroxymethyl- and methoxy-derivatives at positions C1, N2, C9, and C11	[98,99,100]
CYP1A2	Ellipticine	Detoxification	7-hydroxyellipticine, 9-hydroxyellipticine	[96]
	Olivacine	Activation	hydroxy-, hydroxymethyl- and methoxy-derivatives at positions C1, N2, C9, and C11	[98,99,100]
	Etoposide	Activation	Etoposide catechol	[132]
CYP2A6	Tegafur	Activation	5-fluorouracil	[119]
CYP2B6	Cyclophosphamide	Activation	4-hydroxycyclophosphamide	[150]
	Ifosfamide	Activation	4-hydroxyifosfamide	[150]
CYP2C8	Paclitaxel	Inactivation	6α-hydroxylation	[157]
	Masitinib	Activation	*N*-desmethyl masitinib	[168]
CYP2C9	Cyclophosphamide	Activation	4-hydroxycyclophosphamide	[151]
CYP2C19	Tamoxifen	Activation	4-hydroxytamoxifen	[105]
	Thalidomide	Activation	5-hydroxythalidomide	[118]
CYP2D6	Ellipticine	Activation	Ellipticine N2-oxide	[96]
	Tamoxifen	Activation	N-desmethyltamoxifen, 4-hydroxytamoxifen, endoxifen (4-hydroxy-N-desmethyltamoxifen)	[105,106]
	Masitinib	Activation	*N*-desmethyl masitinib	[168]
CYP2E1	Etoposide	Activation	Etoposide catechol	[132]
CYP3A4/ CYP3A5	Ellipticine	Activation, DNA adducts formation	13-hydroxyellipticine and ellipticine N2-oxide	[96]
	Tamoxifen	Activation	N-desmethyltamoxifen, endoxifen. alpha-hydroxytamoxifen	[105]
	Flucloxacillin	Inactivation	5′-hydroxymethylflucloxacillin	[131]
	Etoposide	Activation	Etoposide catechol	[132]
	Paclitaxel	Inactivation	Hydroxy- and dihydroxy-derivatives	[135,136]
	Docetaxel	Inactivation	Hydroxy- and dihydroxy-derivatives	[135,137]
	Cyclophosphamide	Inactivation	4-hydroxycyclophosphamide	[150]
	Vincristine	Activation	Desacetylvincristine, N-oxide, epoxides	[162]
	Vinblastine	Activation	Desacetylvinblastine, N-oxide, epoxides	[161]
	Imatinib	Activation	N-desmethylimatinib	[163]
	Masitinib	Activation	N-desmethyl masitinib	[168]

**Table 2:** Statistically significant *CYP* gene polymorphisms involved in cancer in various populations.

Polymorphism	Population	Biological Effect	Cancer Type	References
CYP3A4*22, CYP3A5*3	N/A	Docetaxel poor metabolizing phenotype, grade 3/4 adverse events	Breast cancer	[138]
CYP1B1*3 CG	Mexican	Positive association with cancer risk	Breast cancer	[196]
*CYP17* TC	Chinese	Positive association with cancer risk	Breast cancer	[199]
*CYP19* (TTTA)_10_	Brazilian	Positive association with cancer	Breast cancer	[201]
*CYP17* A1 *CYP19* TT	Turkish	The combination of two *CYP* genes alleles is protective for BC patients	Breast cancer	[202]
*CYP1B1* N453S	N/A	Decreased cancer risk	Endometrial cancer	[203]
*CYP1B1* R48G	Polish	Increased risk of cancer	Endometrial cancer	[204]
*CYP1A1* AA	Mediterranean	Increased risk of cancer	Endometrial cancer	[205]
*CYP1B1* rs1056836	Caucasian, Asian	Increased risk of cancer	Ovarian cancer	[208]
*CYP17*TC	Japanese	Increased risk of cancer	Prostate cancer	[248]
*CYP1A1* GA+GG, *CYP1A2* CA+AA	Japanese	Increased risk of cancer among individuals with the NAT2 slow acetylator	Prostate cancer	[250]
*CYP1B1* A119S	Caucasian	Increased risk of cancer	Prostate cancer	[253]
*CYP1B1* rs1048943, *CYP1B1* rs4646903	Asian	Increased risk of cancer	Prostate cancer	[257]
CYP3A4*1B	Caucasian	Increased risk of cancer	Prostate cancer	[258]
CYP3A5*3	African	Increased cancer risk	Prostate cancer	[259]
CYP3A4*1B, CYP3A5*3	N/A	Increased cancer risk	Prostate cancer	[260]
CYP3A5*3/*3	N/A	Increased overall survival in patients with metastatic cancer	Prostate cancer	[261]
CYP2D6*10	N/A	Less frequent in cancer patients	Hepatocellular carcinoma	[290]
CYP2A6*4A	Japanese	Significant association with cancer	Hepatocellular carcinoma	[291]
*CYP2D6* TT	N/A	Decreased risk of cancer	Hepatocellular carcinoma	[292]
*CYP24A1* rs6013897	N/A	Predictor of liver cirrhosis and cancer	Hepatocellular carcinoma	[293]
CYP2E1*5B, CYP2E1*6	N/A	Inversed cancer risk	Hepatocellular carcinoma	[295]

## Data Availability

No new data were created or analyzed in this study.
